# Epizootic to enzootic transition of a fungal disease in tropical Andean frogs: Are surviving species still susceptible?

**DOI:** 10.1371/journal.pone.0186478

**Published:** 2017-10-17

**Authors:** Alessandro Catenazzi, Andrea Swei, Jacob Finkle, Emily Foreyt, Lauren Wyman, Vance T. Vredenburg

**Affiliations:** 1 Department of Zoology, Southern Illinois University, Carbondale, Illinois, United States of America; 2 Department of Biology, San Francisco State University, San Francisco, California, United States of America; 3 Department of Biology, Gonzaga University, Spokane, Washington, United States of America; 4 Department of Ecology and Evolutionary Biology, Princeton University, Princeton, New Jersey, United States of America; Universitat Zurich, SWITZERLAND

## Abstract

The fungal pathogen *Batrachochytrium dendrobatidis* (Bd), which causes the disease chytridiomycosis, has been linked to catastrophic amphibian declines throughout the world. Amphibians differ in their vulnerability to chytridiomycosis; some species experience epizootics followed by collapse while others exhibit stable host/pathogen dynamics where most amphibian hosts survive in the presence of Bd (e.g., in the enzootic state). Little is known about the factors that drive the transition between the two disease states within a community, or whether populations of species that survived the initial epizootic are stable, yet this information is essential for conservation and theory. Our study focuses on a diverse Peruvian amphibian community that experienced a Bd-caused collapse. We explore host/Bd dynamics of eight surviving species a decade after the mass extinction by using population level disease metrics and Bd-susceptibility trials. We found that three of the eight species continue to be susceptible to Bd, and that their populations are declining. Only one species is growing in numbers and it was non-susceptible in our trials. Our study suggests that some species remain vulnerable to Bd and exhibit ongoing population declines in enzootic systems where Bd-host dynamics are assumed to be stable.

## Introduction

The eastern slopes of the Andes have one of the highest diversity of amphibians in the world [1]. The recent epizootic (i.e., an epidemic in animal populations) of a pathogenic fungus, *Batrachochytrium dendrobatidis* (Bd; [2]) which causes the fatal disease chytridiomycosis, has caused the widespread collapse of montane Neotropical amphibian communities [3–6]. Although the pathways of disease transmission are still largely unknown, several studies have proposed that the disease may spread in epizootic waves throughout even the most remote areas of Central and South America [4, 7]. The effects on amphibian biodiversity have been devastating [5, 8]. Despite this, many species remain in areas that experienced population declines and extinctions. It is unknown if the surviving species exhibit stable host/pathogen dynamics with Bd and exhibit stable population dynamics, or whether these surviving species are still vulnerable to disease outbreak and population collapse.

Chytridiomycosis has confounded disease ecologists and conservation biologists because theory predicts that a pathogen will fade out when its host population is driven below a minimum threshold density [9, 10]. Yet there are numerous examples of Bd invasions that resulted in population extirpations and species extinctions [5, 11, 12]. In outbreak areas, highly susceptible species succumb quickly [12], while less or non-susceptible species remain and are assumed to be safe [13]. The mechanisms leading to survival are still not well understood; however, it is assumed that the host/Bd dynamics transition from an epizootic dynamic state [12] to a more stable enzootic state [14] when most susceptible species have been extirpated. For some host populations, the host/Bd dynamics have transitioned from epizootic to enzootic states after suspected declines [14, 15], but because declines at these sites occurred before Bd was described, details of the host/Bd dynamics can only be surmised. Some host populations remain stable in spite of the host/Bd dynamics being enzootic, because demographic processes such as recruitment can compensate for mortality of infected adults [15–17]. Clearly both conservation and theory would benefit from a better understanding of the factors that drive the transition from epizootic to enzootic states and allow survival of some species [18, 19]. Recent studies hint at the role of amphibian reservoir hosts for population declines in endemically infected species [19, 20], and the role of environmental conditions that are unfavorable to Bd for promoting population recovery [19].

Peru has some of the highest amphibian diversity of any region (e.g. 562 species, 303 endemic, AmphibiaWeb 2017). The earliest records of chytridiomycosis in Peru [21] are from Bd-infected harlequin toads dying en masse in northern Peru in 1999 [22], followed by a 2002 Bd-caused die-off of *Telmatobius marmoratus* in southern Peru [23]. These are isolated single species examples, but we have previously demonstrated that Bd emergence between 2000 and 2007 was linked with the collapse of the amphibian community in Manu National Park in southern Peru [5, 24]. To address species trajectories after the initial epizootic, we returned to a highly diverse (55 species, 1200–3800 m) Peruvian amphibian community ten years after a major epizootic event [5]. The Bd-caused collapse of amphibian communities in the tropical Andes described by Catenazzi et al. (2011) appeared to stabilize by 2009, thus we hypothesized that in 2012, surviving host species display enzootic host/pathogen dynamics.

We hypothesized that after more than a decade of Bd invasion and host declines, Bd susceptible hosts would be uncommon or extirpated, and thus community-wide Bd epizootics may no longer occur in this area. Host/Bd dynamics may have transitioned to a more stable state (i.e., to an enzootic state) in remaining amphibian communities. In this study we provide field data on wild populations before and after the mass declines. We measure Bd prevalence and infection intensities to explore Bd dynamics in eight surviving amphibian species. We use in situ susceptibility trials to test whether eight species that persisted post mass declines differ in their ability to survive when exposed to Bd.

The eight focal species we selected exhibit a range in habitat specialization, elevational distribution, and reproductive mode. We selected the gladiator frog (*Hypsiboas gladiator)*, an aquatic-breeding species with a tadpole phase (i.e. non-direct developer), two species of direct developing (i.e. no tadpole phase) terrestrial marsupial frogs (*Gastrotheca excubitor* and *G*. *nebulanastes*) and five species of terrestrial direct developing frogs (*Psychrophrynella usurpator*, *Pristimantis danae*, *P*. *pharangobates*, *P*. *platydactylus* and *P*. *toftae*). We also surveyed populations of these focal species before and after the outbreak to quantify the demographic response to the outbreak. We collected Bd infection prevalence and intensity estimates, important factors used to describe disease host/Bd dynamics [12, 14, 25]. As a first step towards understanding the impact of Bd on this community, we conducted Bd susceptibility trials *in situ* on the eight focal species. We compare our experimental results to population trends and infection dynamics in the wild, and explored whether there was any association between species susceptibility to Bd and change in population abundance before and after the epizootic.

## Materials and methods

### Ethics statement

This work has been approved by the Animal Care and Use Committees of San Francisco State University (Protocol #A12-07) and Southern Illinois University (Protocol #13–027), and permits to carry on this research have been issued by the Peruvian Ministry of Agriculture. The Asociación para la Conservación de la Cuenca Amazónica authorized work at its Wayqecha Biological Station.

### Study site

**Fig 1:**
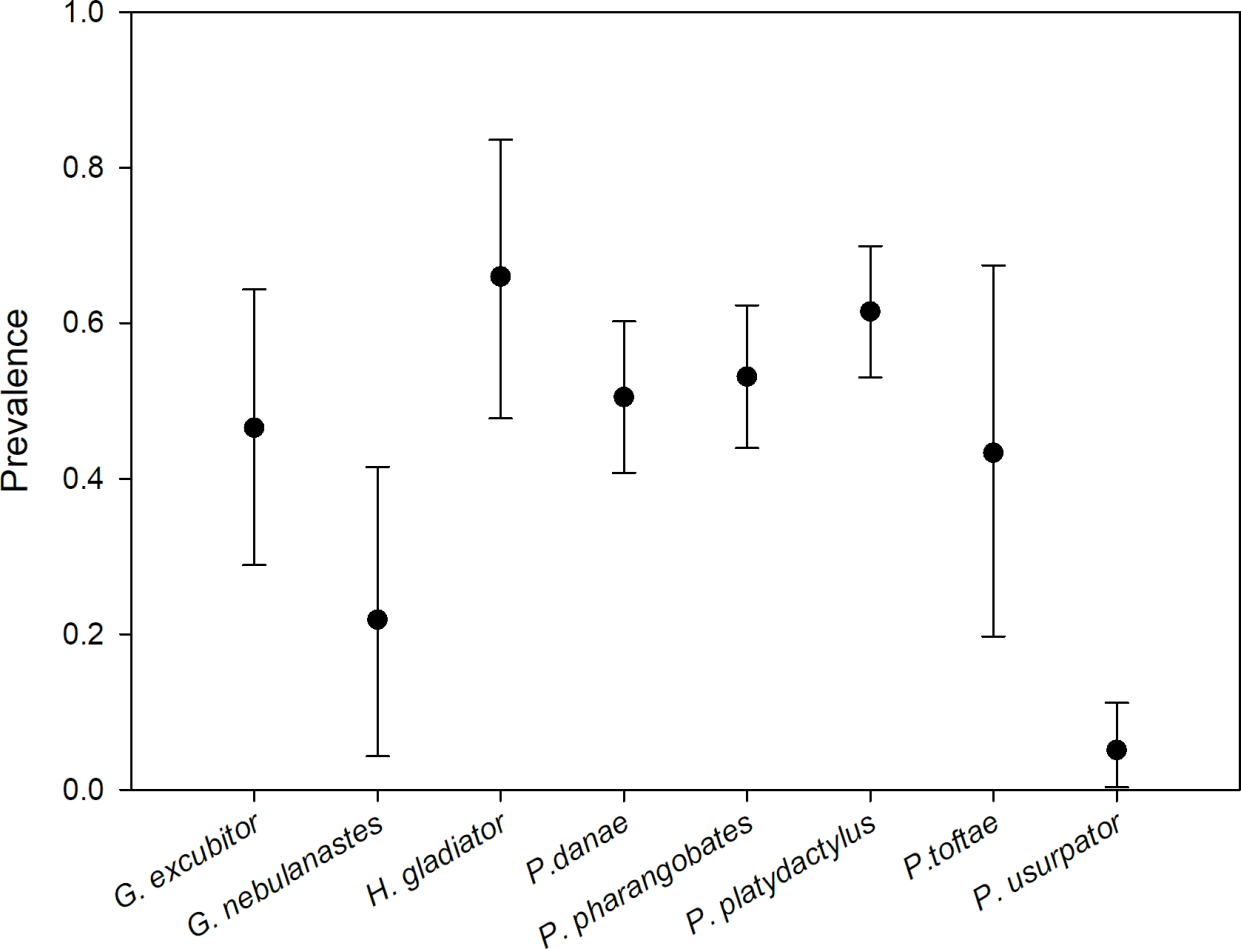
Variation in Bd infection prevalence for eight species of frogs in the montane forests of Manu National Park, Peru. Frogs were sampled during the dry season of 2012, approximately a decade after the Bd epizootic caused a collapse of montane amphibian communities.

Our study took place in the Kosñipata Valley adjacent to Manu National Park near Cusco, southeastern Peru [26]. Manu National Park includes 17,163 km^2^ of largely undisturbed Amazonian lowlands and Andean mountains from 300 m to 4020 m in elevation. In this study, we sampled habitats continuously from 1200 m to 3800 m, and captured frogs in montane forests, scrub, and high-Andean grasslands (Fig 1). Before the chytridiomycosis epizootic, 55 species occurred in this elevational range; after the outbreak, 19 species disappeared [5, 24].

### In situ Bd susceptibility trials

We conducted susceptibility trials in rustic conditions at the remote Wayqecha Biological Field station located in our study area [5, 27]. We collected 122 frogs from the eight focal species for the susceptibility trials. We hand-captured frogs and placed them individually in plastic bags until they were placed in experimental chambers. All animals were swabbed on the skin for Bd, measured (SVL), weighed (g), and sexed. Because the study area contains Bd, we immersed all study animals in a 1% itraconazole solution for 5 minutes a day for seven consecutive days before susceptibility trials began [28]. After the anti-fungal procedure, each animal was randomly assigned into either the treatment group (Bd exposed) or the control group (no Bd exposure). We used 78 Bd-infected *Telmatobius marmoratus*, a highly Bd-infected species native to the study area, to expose our study species to Bd, because fungal cultures could not be maintained at the remote field station. Infected *T*. *marmoratus* were purchased from a live frog stand at the San Pedro market in Cusco, Peru. We purchased 16–20 live *T*. *marmoratus*, on four different dates (22 June, 3, 18 and 23 July 2012). In previous studies we showed that animals from this market stand were all highly infected with Bd [29, 30]. Since we did our Bd susceptibility trials *in situ*, we analyzed Bd swabs after the end of the entire experiment.

Animals in the susceptibility trials were either exposed to *T*. *marmoratus* Bd (treatment group, Bd exposed) or not exposed to *T*. *marmoratus* Bd (control group, not Bd exposed). The treatment group frogs (Bd exposed) were temporarily placed in a two-chambered exposure container with a single Bd-infected *T*. *marmoratus*. The exposure time was 5 hours per day for 5 consecutive days. Animals in the control group were not exposed to *T*. *marmoratus* because all *T*. *marmoratus* were Bd positive. The number of animals in the control group (not Bd exposed) ranged from 4–7 individuals depending on the species, while the number of animals in the treatment group (Bd exposed) ranged from 4–18 individuals, depending on the species. Exposure containers were 1.2 L plastic containers with two parallel mesh barriers affixed across the middle of the container. The mesh barriers created two equal-sized chambers within the exposure container. The mesh barriers were 1 cm apart from each other, preventing direct contact between the treatment frog and the Bd-infected *T*. *marmoratus*, but allowing water to flow freely between the two sides. We added 100 ml of water to each exposure container. We examined each exposure container visually every 10 min during each 5 hour exposure period to ensure that frogs stayed in contact with the water. Bd exposed individuals were exposed to the same *T*. *marmoratus*, unless the infected *T*. *marmoratus* died during the exposure period. When this occurred, the *T*. *marmoratus* was immediately replaced by another individual of the same species. Some *T*. *marmoratus* were used in multiple exposure sessions, but none were used in more than three sessions.

To determine Bd-infection status of our experimental animals, we collected skin swabs for each study animal at the time of capture, immediately following anti-fungal treatment, and once a week throughout the length of the experiment. For the treatment group (Bd exposed) and all *T*. *marmoratus*, we collected a skin swab immediately before and after the five-day exposure period. Skin swabs were analyzed using a standard qPCR protocol [31]. Body weight and SVL was recorded for each animal at the time of capture, immediately following the anti-Bd treatment, and at weekly intervals throughout the experiment.

Control frogs (not Bd exposed) were housed individually in 1.2 L plastic containers with vented isolation tops (lids). We could not expose them to uninfected *T*. *marmoratus* because all *T*. *marmoratus* were infected, but control frogs were kept in the same type of containers and under similar conditions as the Bd exposed individuals during the exposure period. The experiment began 14 June 2012 and ended 26 August 2012. All experiments were run under ambient conditions (they were outside) so that frogs experienced natural variations in temperature and photoperiod during the experiment. Two wet paper towels were placed in each container. Frogs were fed daily with locally captured invertebrates. Frogs that died during the anti-fungal treatment or during the Bd exposure period were excluded from the analyses. We formalin-preserved deceased individuals following standard museum protocols [32] and deposited the specimens in the herpetological collection of Centro de Ornitología y Biodiversidad (CORBIDI) in Lima, Peru. We previously reported survivorship data for *G*. *excubitor* and *G*. *nebulanastes* [27], but because this study did not include population abundance data, for sake of simplicity we include these data here. Data for all species are reported in S1 Table.

### Relative abundance, infection prevalence and intensity in wild populations

We used 10x10 m^2^ leaf-litter plots and visual encounter surveys to estimate frog species abundances on the basis of species-specific habitat preferences and life history traits. These data were collected during the wet seasons of 1998–1999 (before epizootic) and during the wet seasons of 2008–2009 (after epizootic, [5]) throughout the elevational range of each species, minimizing the importance of other seasonal and local factors affecting intra- and inter-annual variation in population abundance. Albeit climate may also have caused changes in abundance between the sampling years, our two recent sampling seasons differed sharply in precipitation [5], such that our after-epizootic data combine relative abundance from a range of climatic conditions. Leaf-litter plots (four plots for every 100 m change in elevation) were used to estimate relative densities (frogs/ha) for *G*. *excubitor*, *Psychrophrynella usurpator*, *Pristimantis danae* and *P*. *pharangobates*, while visual encounter surveys (covering the entire elevational range) estimated number of individuals per person-hour for *G*. *nebulanastes*, *H*. *gladiator* and the four species of *Pristimantis*. We calculated an index of population trends as the ratio between the average of the 2008–2009 estimates and the 1998–1999 estimates (1 = no change). For each species sampled in previous population surveys, we collected skin swabs to detect the presence and infection intensity of Bd from May–October 2012 (dry season) while conducting susceptibility trials. We calculated Bd infection prevalence by dividing the number of infected animals by the total number of animals assayed per species (for infection intensity, see below). Bd infection data are available online at the Amphibian Disease database (https://n2t.net/ark:/21547/AXY2).

### Molecular methods

To measure Bd infection prevalence and intensity, the skin of each frog was swabbed with a sterile rayon-tipped swab (Medical Wire & Equipment MW113). Swabs were gently stroked across the skin a total of 30 times per frog: 5 strokes on each side of the abdominal midline, 5 strokes on the inner thighs of each hind leg, and 5 strokes on the foot webbing of each hind leg. This technique is widely used and does not harm the animals [33].

We used a quantitative Polymerase Chain Reaction (qPCR) assay to detect Bd and to quantify the intensity of Bd-infection [31]. We followed standard DNA extraction and real-time PCR methods [31], except that we analyzed single-swab extracts once instead of in triplicate [33]. Swabs were extracted using PrepMan Ultra and analyzed using the 7300 Real-Time PCR System (Life Technologies, Carlsbad, CA) at San Francisco State University. The qPCR assay estimates Bd genomic equivalents (GE) in each sample and we convert this to provide “zoospore equivalents” on each frog [12, 14] to allow for comparison between studies.

### Statistical analyses

We calculated Bd prevalence (proportion of swabbed frogs infected with Bd) by considering swabs as Bd-positive if zoospore equivalents > 0 and Bd-negative if zoospore equivalents = 0. Only species or groups with at least five swabbed individuals were considered for analyses of prevalence and only groups with at least five positive individuals were considered for analyses of infection intensity. We report prevalence data using Bayes credible intervals [34].

We used the *Survival Package* in R 2.15.2 to compare survivorship of control (not Bd exposed) and treatment frogs (Bd exposed). We used Cox’s proportional hazards model with censoring [35, 36] to assess the risk of dying for treatment frogs (Bd exposed). For all eight species we performed separate analyses of covariance with initial weight as the continuous explanatory variable and treatment group (control, treatment) as the categorical explanatory variable. We hypothesized that initial weight could have affected survival during the experiments. However, in none of the species initial weight affected survival, and thus weight was subsequently removed from the models.

We compared the relative abundances of the eight species of frogs by using generalized linear mixed effects model with Poisson errors. For leaf litter plots, we used the number of frogs found within each 100 m^2^ plot. Relative abundances for visual encounter survey data were standardized by search effort, because duration and number of observers varied among surveys, and these data were rounded to integer prior to analysis. We conducted separate analyses for leaf-litter plots and visual encounter survey data. We only made within-species comparisons because abundance data were restricted to the unique elevational range of each species. Infection intensities were log-transformed (base 10) prior to analyses. We used non-parametric tests (Wilcoxon rank sum, Kruskal-Wallis) to compare maximum infection intensity between control (no Bd exposure) and treatment (Bd exposed) individuals, because there was substantial overdispersion in the data, and sample sizes were small. Averages are followed by the standard error.

## Results

### Bd prevalence and intensity of infection in the field

Field sampling in 2012 found Bd infection in all species within our study area (Fig 1, Table 1), but prevalence varied among species (chi-square = 55.7, df = 7, p < 0.001) and exceeded 60% in the two riparian species, *Hypsiboas gladiator* and *Pristimantis platidactylus*. Infection intensities ranged from 0.01 to 26,208.80 ZE, but did not differ among species (F_7,218_ = 0.47, p = 0.853; Table 1). Among infected frogs, most (63.1%) had low to moderate infection intensities: 32.7% had 1–9.99 ZE, 17.1% had 10–99.9 ZE, and 13.3% had 100–999.9 ZE. Two individuals of *H*. *gladiator* had infection intensities nearly reaching 10,000 ZE, and one individual of *G*. *excubitor* had the highest intensity of 26,208.80 ZE.

**Table 1:** Variation in prevalence and intensity of infection (log-transformed zoospore equivalents, ZE) in eight species of tropical montane frogs in the Peruvian Andes during May–October 2012.

Species	N	Prevalence	Infected	ZE ±SE (range)
*Gastrotheca excubitor*	28	0.47 (0.29–0.64)	13	2055 ± 2013 (1–26209)
*Gastrotheca nebulanastes*	15	0.22 (0.04–0.42)	3	21 ± 9 (4–31)
*Hypsiboas gladiator*	24	0.66 (0.48–0.84)	16	82 ± 57 (1–922)
*Pristimantis danae*	99	0.51 (0.41–0.60)	50	51 ± 26 (1–221)
*P*. *pharangobates*	111	0.53 (0.44–0.62)	59	26 ± 7 (1–247)
*P*. *platydactylus*	125	0.62 (0.53–0.70)	77	91 ± 70 (1–5333)
*P*. *toftae*	14	0.43 (0.20–0.67)	6	211 ± 207 (1–1247)
*Psychrophrynella usurpator*	48	0.04 (0.00–0.11)	2	5 ± 3 (1–8)

### Bd susceptibility trials

**Fig 2:**
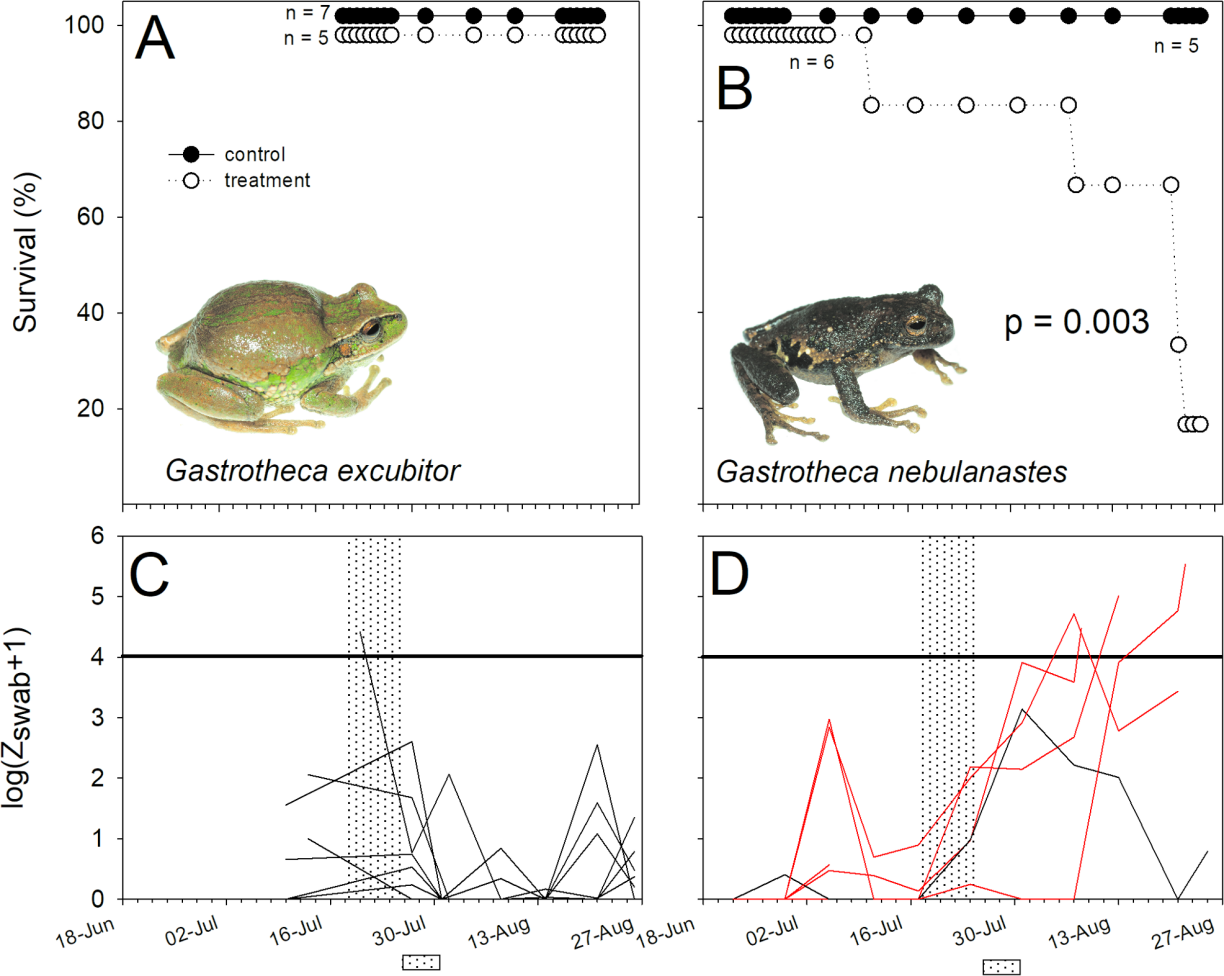
Differences in survival and Bd infection intensity in two species of marsupial frogs *Gastrotheca* (Hemiphractidae). (A, B) Percentage survival, and (C, D) variation in log-transformed zoospore equivalents (Bd infection intensity) of Bd-exposed individuals of *Gastrotheca excubitor* (left panels) and *G*. *nebulanastes* (right panel). The shaded band represents the period of daily immersion in itraconazole solution to clear infection, while the shaded box represents the period of experimental exposure to Bd. Red lines represent individuals that died during the trial.

**Fig 3:**
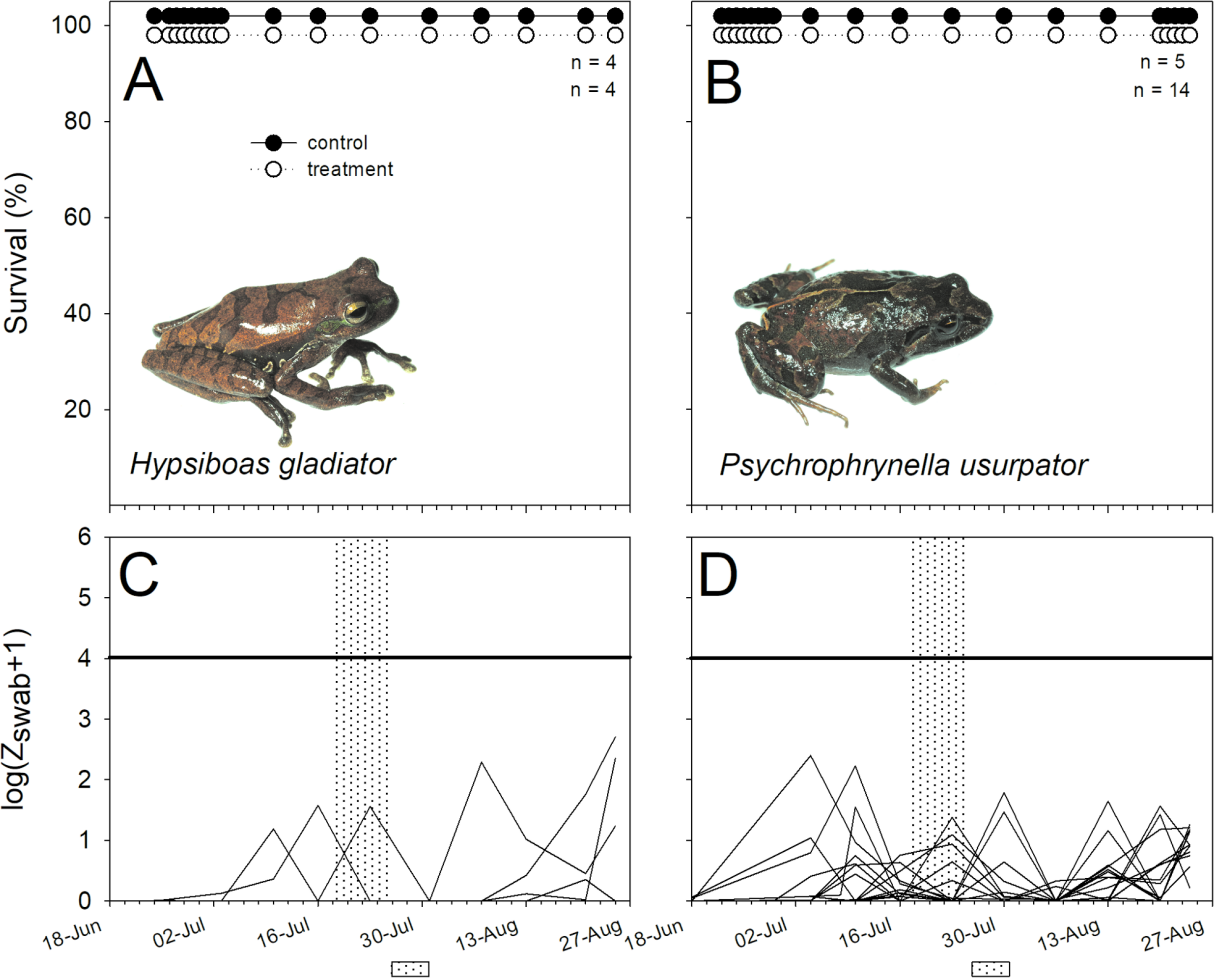
The gladiator frog *Hypsiboas armatus* (Hylidae) and the Cusco Andes frog *Psychrophrynella usurpator* (Craugastoridae) survived experimental infection. (A, B) Percentage survival, and (C, D) variation in log-transformed zoospore equivalents (Bd infection intensity) of Bd-exposed individuals of *Hypsiboas gladiator* (left panels) and *Psychrophrynella usurpator* (right panel). See Fig 2 for caption details.

**Fig 4:**
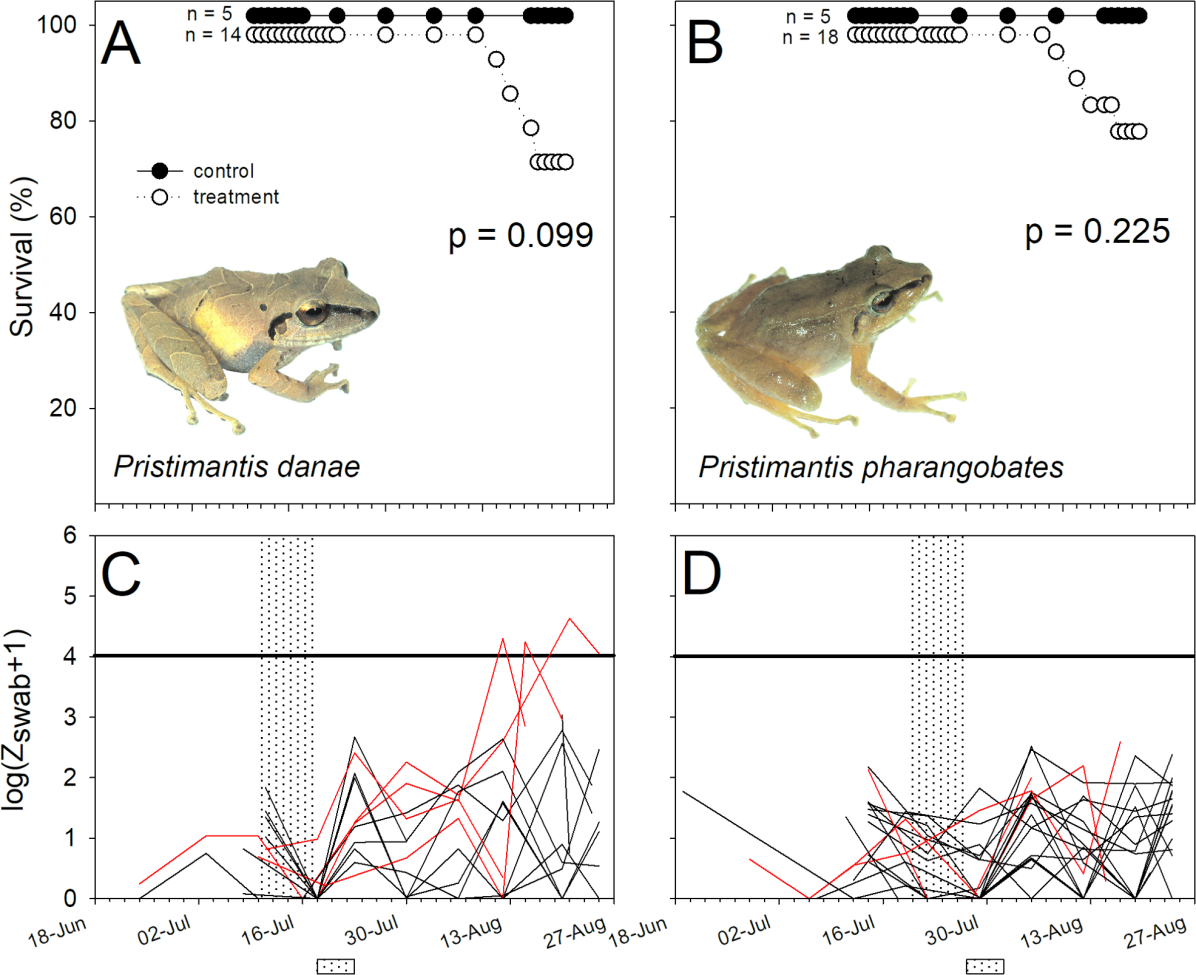
Differences in survival and Bd infection intensity in two non-susceptible species of terrestrial-breeding frogs, *Pristimantis danae* and *Pristimantis pharangobates* (Craugastoridae). (A, B) Percentage survival, and (C, D) variation in log-transformed zoospore equivalents (Bd infection intensity) of Bd-exposed individuals of *Pristimantis danae* (left panels) and *P*. *pharangobates* (right panel). See Fig 2 for caption details.

**Fig 5:**
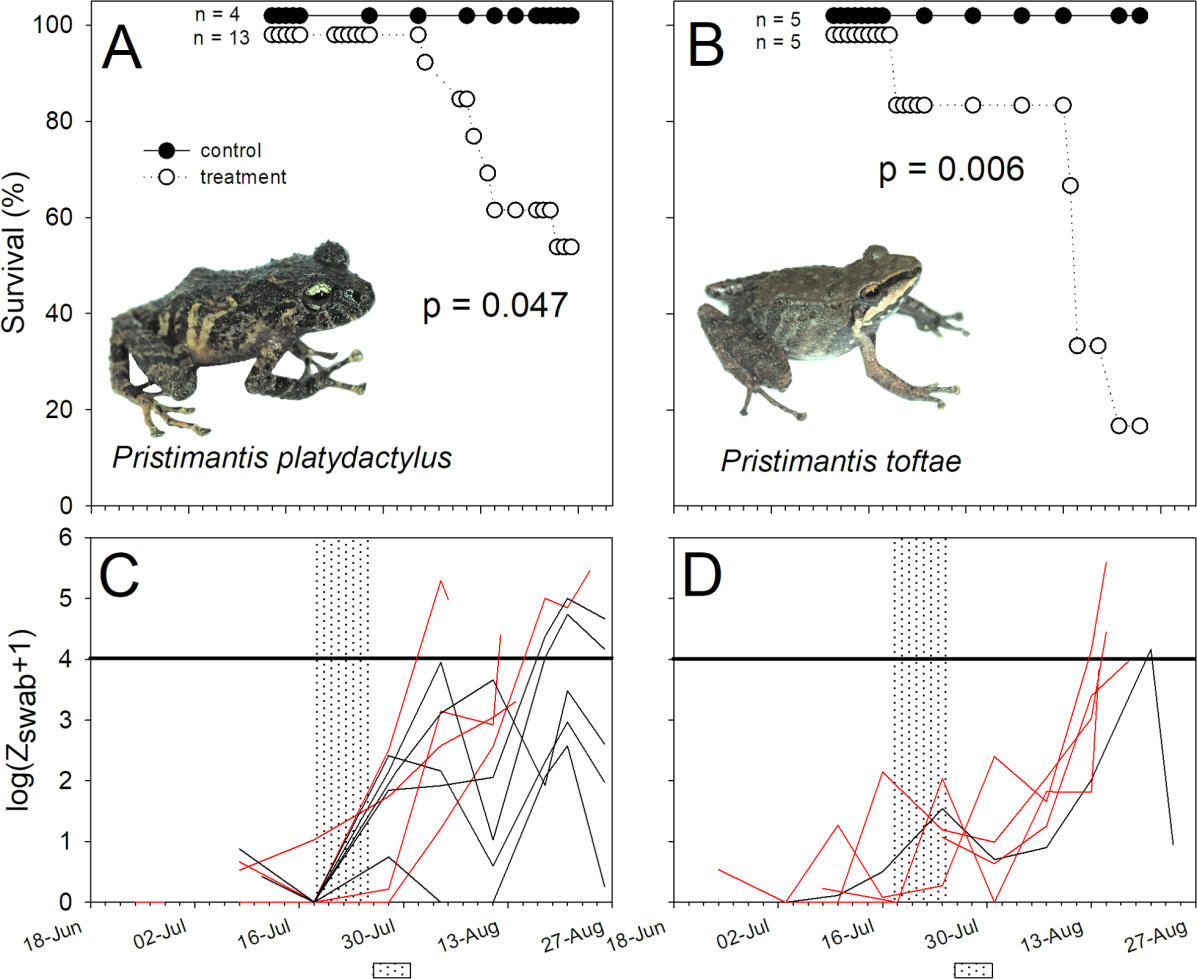
Differences in survival and Bd infection intensity in two susceptible species of terrestrial-breeding frogs, *Pristimantis platydactylus* and *Pristimantis toftae* (Craugastoridae). (A, B) Percentage survival, and (C, D) variation in log-transformed zoospore equivalents (Bd infection intensity) of Bd-exposed individuals of *Pristimantis platydactylus* (left panels) and *P*. *toftae* (right panel). See Fig 2 for caption details.

Bd infection intensities on the Bd source, *T*. *marmoratus*, used in this experiment were high, averaging 2655.65 ± 795.15 ZE at the time of the susceptibility experiment. Susceptibility to Bd exposure varied considerably between our focal species (Table 2, Figs 2–5). Bd exposure significantly lowered the survival of *G*. *nebulanastes*, *P*. *platydactylus* and *P*. *toftae* (Figs 2–4), when compared to unexposed control frogs, but not in the other five species tested (Table 2, Figs 2–5). In the three susceptible species, the level of infection intensity (at last day of experiment) differed significantly between treatment (Bd exposed) and control groups (not Bd exposed): 83,453.41 ± 63,766.95 vs. 0.33 ± 0.20 ZE in *G*. *nebulanastes* (t = -3.75, df = 5.05, p = 0.013), 36,377.01 ± 22,146. 71 vs. 45.27 ± 24.69 ZE in *P*. *platydactylus* (t = -2.93, df = 12.56, p = 0.012), and 87,499 ± 76,715.20 vs. 185.75 ± 175.23 ZE in *P*. *toftae* (t = -2.79, df = 7.14, p = 0.026). Infected frogs exhibited symptoms of chytridiomycosis (abnormal skin shedding, tetanic spasms, loss of righting reflex; see videos in S1 File) prior to death.

**Table 2:** Survival analysis for Bd susceptibility trials of eight species of tropical montane forest frogs subjected to control or experimental treatment. Population ratios are calculated from relative abundances of populations before and after the epizootic using leaf litter plots (plot) and visual encounter surveys (VES) in 1998–1999 and 2008–2009. Treatments with no mortality are indicated with NA’s for *P*-value and days to death. Population ratios provided are based on visual encounter surveys except for cryptic species that were assessed using leaf litter plot surveys indicated by ^L^.

Species	N		*P*	Duration (days)	Days to death (mean)		Pop. ratios
	control	treatment			control	treatment	
Hemiphractidae							
*G*. *excubitor*	7	5	*NA*	26	*NA*	*NA*	0.54^L^
*G*. *nebulanastes*	5	6	**0.003**	51	*NA*	36.2	**0.07**
Hylidae							
*H*. *gladiator*	4	4	*NA*	47	*NA*	*NA*	0.51
Strabomantidae							
*Pr*. *danae*	5	14	0.099	33	*NA*	27.3	0.66^L^
*Pr*. *pharangobates*	5	18	0.225	28	*NA*	20.7	0.59^L^
*Pr*. *platydactylus*	4	13	**0.047**	29	*NA*	16.3	0.45
*Pr*. *toftae*	5	5	**0.006**	32	*NA*	22.5	0.51
*Ps*. *usurpator*	5	14	*NA*	52	*NA*	–	2.24^L^

^L^ Indicates populations surveyed with leaf litter plots.

Significant *P*-values are in bold.

Phylogenetic relatedness and life-history characteristics did not explain susceptibility to elevated exposure to Bd (Table 2). For example, two closely related direct-developing marsupial species in the genus *Gastrotheca* showed different susceptibility to Bd; *G*. *nebulanastes* was highly susceptible while *G*. *excubitor* was not (Fig 2). Similarly, ground-dwelling direct-developing species in the genus *Pristimatis* also differed in susceptibility: *P*. *platydactylus* and *P*. *toftae* were susceptible (Fig 5), whereas *P*. *danae* and *P*. *pharangobates* were not (Fig 4).

There was no difference in survival between treatment and control animals in *P*. *danae*, but treatment individuals that died had higher infection intensity (Fig 4), consistent with Bd susceptibility (e.g. died = 6,023.33 ± 3,343.28 ZE, vs. control frogs = 16.34 ± 11.52 ZE; t = -5.04, df = 7.998,p = 0.001). Despite exposure to high levels of zoospores, no mortality occurred in three study species, *G*. *excubitor* (Fig 2), *H*. *gladiator* and *P*. *usurpator* (Fig 3, Table 1), and in these species infection intensity in treatment animals (Bd exposed) remained below 1,000 ZE throughout the experiment (S1 Table).

### Relative abundances before vs. after the epizootic

**Fig 6:**
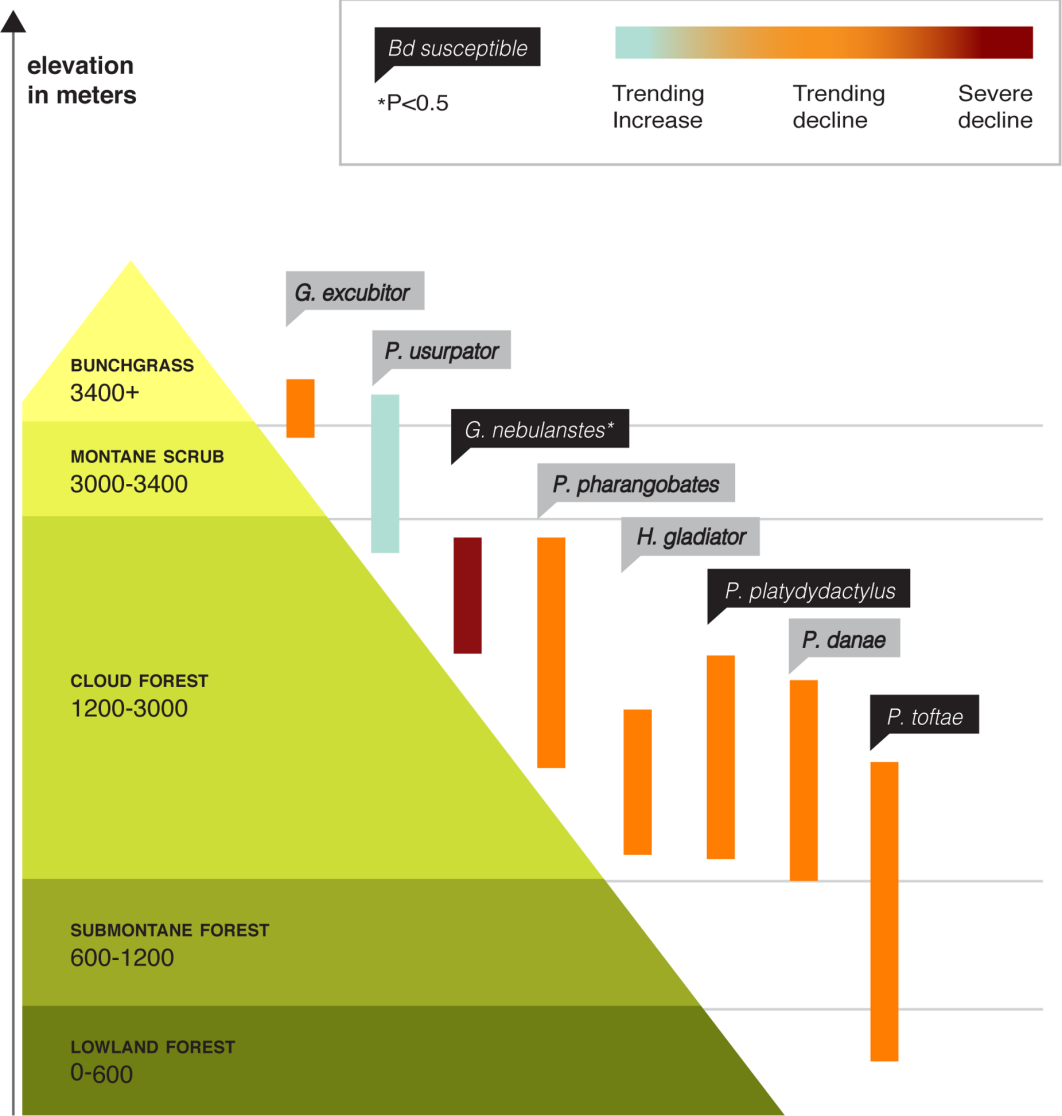
Elevational ranges and summary of findings for the species examined in this study. The eight species examined in this study are among the 36 species surviving out of originally 55 species distributed from 1200 to 3800 m prior to the Bd epizootic. The mountain shown is the Amazonian slope of the Cordillera de Paucartambo, Cusco, near Manu National Park, Peru. Species found to be susceptible to Bd are indicated and population survey results from pre and post-epizootic are shown.

The ratio of relative abundances before the epizootic (1998–1999) compared to after the epizootic (2008–2009) was <1 for all species (except for *P*. *usurpator*) suggesting population declines (Fig 6; Table 1; S2 Table). These results indicate that the Bd epizootic caused the frog population declines, and that frog populations did not recover during the first decade after the epizootic.

## Discussion

Amphibians are experiencing a mass extinction event [37] caused in part by the emergence of a lethal fungal pathogen (*Batrachochytrium dendrobatidis*, Bd). The disease-induced losses have been substantial (e.g. [8, 38]), yet some species survive the epizootic and are assumed to be non-susceptible. However, because the pathogen remains in these systems after an epizootic, the level of susceptibility of surviving hosts has not been explicitly measured. Understanding host/pathogen disease dynamics is these systems is essential for the management and monitoring of surviving amphibian species following mass declines.

Several studies have shown that infection intensities are directly tied to survival outcomes in wild populations [12, 14]. Our findings support the hypothesis that hosts with high Bd infection intensities (>10,000 zoospore) have reduced survivorship [25]. Sustained, low Bd infection intensity has been found in host populations where host/Bd dynamics are stable [14], while high intensity infections often associate with continued mortality [12, 39]. Comparisons of temporal trends in Bd infection intensity of surviving hosts in the wild may help predict future host population stability. Rising Bd infection intensities may signal new outbreaks whereas stable or non-rising infection intensities may signal stable host populations. However, other factors may complicate interpretation of trends in infection intensities; for example occurrence of loss of infection has been shown to vary seasonally [39]. Furthermore, comparisons of Bd infection intensity data must be done cautiously because Bd standards used in quantitative PCR assays may vary in gene copy number [40]; yet, these data can be calibrated between studies based on the qPCR standards used and the gene copy number of the Bd strain infecting amphibians. Disease metrics (intensity and prevalence through time) coupled with population surveys can thus help predict how Bd will affect species in nature. Bd susceptibility trials improve our predictive capabilities regarding Bd effects on host species.

We assessed the effect of increased Bd exposure on survivorship of species that have endured an amphibian mass extinction caused by a Bd epizootic. The epizootic in the Kosñipata Valley of southern Peru caused the loss of 19 of 55 species that occurred from 1200 to 3800m [5]. More than a decade has elapsed since the collapse of these amphibian communities. We examined focal species that continue to exist in the elevational range where Bd caused the highest loss of species. Since existing frogs (i.e., a subset of the 36 species that did not disappear) had survived the community collapse caused by Bd, we expected weak effects, if any, of increased Bd exposure on the focal species. Instead, we found that three of eight species we tested showed significant ongoing susceptibility to increased Bd exposure and our population trend estimates found that seven of our eight species were less abundant after the epizootic compared to our surveys conducted before the epizootic.

Our assessment of species susceptibility to Bd adds to our understanding of how the amphibian community is responding to Bd a decade after the epizootic, but susceptibility alone cannot describe the host/Bd dynamics. Other factors, especially those related to transmission risk and environmental exposure to Bd [5, 41, 42], likely influence persistence and recovery of amphibian populations at our study site. Field observations suggest that since 2014, populations of some species, such as *P*. *toftae*, are now recovering (A. Catenazzi, unpublished data). Although breeding cycles are poorly known for most species, if we assume continuous reproduction throughout the year and short time lag to sexual maturity (within two years) for all species, populations of surviving species could be recovering within 5–10 years unless host/Bd dynamics alternate between enzootic and epizootic stages. Future surveys will quantify the extent of these population recoveries, and more generally the status of populations known to have declined during the first decade after the epizootic.

An important finding of our study is that phylogenetic relationships were poor predictors of species susceptibility to Bd. Our results are consistent with findings from community-level assessments showing that the degree of population loss and decline among species is random with respect to the community phylogeny [3, 5, 8]. Differential susceptibility may reflect species level differences or perhaps differences in prior exposure intensity to Bd, although our experimental findings do not support the latter, immunoprotective hypothesis (see below). We have shown that skin defenses, particularly differences in the proportion of Bd-inhibiting skin bacteria, are associated with difference in susceptibility in the two species of *Gastrotheca* (Fig 2, [27]), but other factors such as thermal sensitivity of immune response may also affect disease outcome [43, 44].

We found that exposure of field-caught frogs to Bd infected *T*. *marmoratus* caused mortality in patterns consistent with death by chytridiomycosis, as supported by symptoms such as frequent skin shedding and tetanic spams observed in highly infected individuals shortly before death (S1 File). Most *T*. *marmoratus* used for infection had high Bd-infection intensities and therefore presumably shed high numbers of zoospores (e.g., ZE ranging from 1,000–10,000). These values are similar to a proposed threshold that causes death in a number of amphibian species [7, 12, 25, 45] (S1 Table). Our study suggests that frog-to-frog heterospecific transmission (with or without direct contact) can lead to chytridiomycosis and death even in species that were assumed to be resistant to Bd.

The itraconazole anti-fungal treatments did not completely remove Bd infections from study animals, as seen in other studies [46]. Thus, frogs in our Bd exposed treatment were not completely free of Bd when they were exposed to highly infected *T*. *marmoratus*. Our anti-fungal treatments did reduce Bd infection intensities on all individuals equally between treatment and control groups. We propose that exposure of animals that already have a low level infection may be more representative of actual dynamics in the wild where Bd is known to remain in systems after epizootics [15, 47].

Our experimental design, and failure to clear infection in all individuals, indirectly provides support to previous suggestions that an immunoprotective effect of previous Bd exposure might not be a common response across all amphibian species [48–51]. Although we ignore the Bd exposure history of our experimental frogs, it is plausible to assume that many frogs at our study site, where infection prevalence can be as high as 90% [24], have previously been exposed to Bd. Among the three susceptible species in our study, *P*. *platydactylus* and *P*. *toftae* live at elevations with high Bd prevalence [24], and Bd prevalence in their populations is high (Table 1; [5]). Therefore, it is likely that the individuals of *P*. *platydactylus* and *P*. *toftae* that we used in our study had been infected with Bd prior to our experiment. Mitigation strategies emphasizing immunization should thus take into consideration inter-host differences in the effectiveness of protective response following immunization or prior exposure to live Bd.

Despite recent advances in our understanding of disease dynamics in chytridiomycosis [12, 14, 52, 53], this disease remains a major threat to amphibian biodiversity [37]. Our study was conducted in a montane amphibian community 10 years after a Bd-epizootic eliminated 19 of 55 species. Some species fared worse than others. For example, over half of the stream-breeding species disappeared following the arrival of Bd [5]. We might have predicted that aquatic species would be at highest risk, but we would not necessarily have predicted that a terrestrial marsupial frog (*Gastrotheca nebulanastes*) would be susceptible (Table 1). Our *a priori* expectation was that phylogenetic relatedness would predict Bd susceptibility because two genera of frogs (*Atelopus* and *Telmatobius*) disappeared completely from Kosñipata Valley during the Bd epizootic, but phylogenetic relatedness does not appear to be an important predictor for susceptibility. Our Bd susceptibility trials demonstrate that chytridiomycosis continues to be a threat even for some species that survived an initial Bd-caused mass extinction and are now in environments that contain carrier hosts infected with Bd, similar to results from other systems [19, 20, 54]. More importantly, our results suggest that after an epizootic, Bd can still impact amphibian populations and may be regulating their populations at levels lower than pre-epizootic conditions. Our field-based empirical approach provides predictive information that can help determine priority species for conservation action even in regions where epizootics have previously decimated amphibian diversity and abundance.

## Supporting information

S1 Table Raw data for susceptibility trials. This is the S1 Fig legend.
(DOCX)

S1 File Videos showing symptoms of chytridiomycosis in highly infected frogs used in the susceptibility trials. Videos of diseased individuals used during the experiments.
(DOCX)

S2 Table P-values from generalized linear models for change in relative abundances before (1998–1999) and after the epizootic (2008–2009) for leaf litter plots and visual surveys. “–”indicates that no data are available for a given survey and species; Table 2 column refers to data (population ratios and results of GLM analyses) reported in Table 2. When data from both sampling techniques are available, results from leaf litter plots are reported in Table 2 of the manuscript.
(DOCX)

## References

[1] Stuart SN, Chanson JS, Cox NA, Young BE, Rodrigues ASL, Fischman DL, et al Status and trends of amphibian declines and extinctions worldwide. Science. 2004;306(5702):1783–6. doi: 10.1126/science.1103538 15486254 10.1126/science.1103538

[2] Longcore JE, Pessier AP, Nichols DK. *Batrachochytrium dendrobatidis* gen et sp nov, a chytrid pathogenic to amphibians. Mycologia. 1999;91(2):219–27.

[3] Berger L, Speare R, Daszak P, Green DE, Cunningham AA, Goggin CL, et al Chytridiomycosis causes amphibian mortality associated with population declines in the rainforests of Australia and Central America. Proceedings of the National Academy of Sciences USA. 1998;95:9031–6. 10.1073/pnas.95.15.9031 PMC21197 9671799

[4] Lips KR, Diffendorfer J, Mendelson JR III, Sears MW. Riding the wave: Reconciling the roles of disease and climate change in amphibian declines. PLoS Biology. 2008;6(3):441–54. doi: 10.1371/journal.pbio.0060072 18366257 10.1371/journal.pbio.0060072 PMC2270328

[5] Catenazzi A, Lehr E, Rodriguez LO, Vredenburg VT. *Batrachochytrium dendrobatidis* and the collapse of anuran species richness and abundance in the upper Manu National Park, southeastern Peru. Conservation Biology. 2011;25(2):382–91. doi: 10.1111/j.1523-1739.2010.01604.x 21054530 10.1111/j.1523-1739.2010.01604.x

[6] Catenazzi A. State of the world's amphibians. Annual Review of Environment and Resources. 2015;40:91–119.

[7] Cheng TL, Rovito SM, Wake DB, Vredenburg VT. Coincident mass extirpation of neotropical amphibians with the emergence of the infectious fungal pathogen *Batrachochytrium dendrobatidis*. Proceedings of the National Academy of Sciences of the United States of America. 2011;108(23):9502–7. doi: 10.1073/pnas.1105538108 21543713 10.1073/pnas.1105538108 PMC3111304

[8] Crawford AJ, Lips KR, Bermingham E. Epidemic disease decimates amphibian abundance, species diversity, and evolutionary history in the highlands of central Panama. Proceedings of the National Academy of Sciences of the United States of America. 2010;107(31):13777–82. doi: 10.1073/pnas.0914115107 20643927 10.1073/pnas.0914115107 PMC2922291

[9] Anderson R, May R. Population biology of infectious diseases: part 1. Nature. 1979;280:361–7. 460412 10.1038/280361a0

[10] de Castro F, Bolker B. Mechanisms of disease-induced extinction. 2005;8(1):117–26.

[11] Lips KR, Brem F, Brenes R, Reeve JD, Alford RA, Voyles J, et al Emerging infectious disease and the loss of biodiversity in a Neotropical amphibian community. Proceedings of the National Academy of Sciences of the United States of America. 2006;103(9):3165–70. doi: 10.1073/pnas.0506889103 16481617 10.1073/pnas.0506889103 PMC1413869

[12] Vredenburg VT, Knapp RA, Tunstall TS, Briggs CJ. Dynamics of an emerging disease drive large-scale amphibian population extinctions. Proceedings of the National Academy of Sciences of the United States of America. 2010;107(21):9689–94. doi: 10.1073/pnas.0914111107 20457913 10.1073/pnas.0914111107 PMC2906868

[13] Reeder NMM, Pessier AP, Vredenburg VT. A reservoir species for the emerging amphibian pathogen *Batrachochytrium dendrobatidis* thrives in a landscape decimated by disease. Plos One. 2012;7(3). doi: 10.1371/journal.pone.0033567 22428071 10.1371/journal.pone.0033567 PMC3299797

[14] Briggs CJ, Knapp RA, Vredenburg VT. Enzootic and epizootic dynamics of the chytrid fungal pathogen of amphibians. Proceedings of the National Academy of Sciences of the United States of America. 2010;107(21):9695–700. doi: 10.1073/pnas.0912886107 20457916 10.1073/pnas.0912886107 PMC2906864

[15] Retallick RWR, McCallum H, Speare R. Endemic infection of the amphibian chytrid fungus in a frog community post-decline. PLoS Biology. 2004;2(11):1965–71. doi: 10.1371/journal.pbio.0020351 15502873 10.1371/journal.pbio.0020351 PMC521176

[16] Tobler U, Borgula A, Schmidt BR. Populations of a susceptible amphibian species can grow despite the presence of a pathogenic chytrid fungus. Plos One. 2012;7(4). doi: 10.1371/journal.pone.0034667 22496836 10.1371/journal.pone.0034667 PMC3320643

[17] Scheele BC, Hunter DA, Skerratt LF, Brannelly LA, Driscoll DA. Low impact of chytridiomycosis on frog recruitment enables persistence in refuges despite high adult mortality. Biological Conservation. 2015;182:36–43. doi: 10.1016/j.biocon.2014.11.032

[18] Scheele B, Guarino F, Osborne W, Hunter DA, Skerratt LF, Driscoll DA. Decline and re-expansion of an amphibian with high prevalence of chytrid fungus. Biological Conservation. 2014;170:86–91. doi: 10.1016/j.biocon.2013.12.034

[19] Scheele BC, Skerratt LF, Grogan LF, Hunter DA, Clemann N, McFadden M, et al After the epidemic: Ongoing declines, stabilizations and recoveries in amphibians afflicted by chytridiomycosis. Biological Conservation. 2017;206:37–46. doi: 10.1016/j.biocon.2016.12.010

[20] Scheele BC, Hunter DA, Brannelly LA, Skerratt LF, Driscoll DA. Reservoir-host amplification of disease impact in an endangered amphibian. Conservation Biology. 2017;31(3):592–600. doi: 10.1111/cobi.12830 27594575 10.1111/cobi.12830

[21] Catenazzi A, von May R. Conservation status of amphibians in Peru. Herpetological Monographs. 2014;28:1–23.

[22] Venegas PJ, Catenazzi A, Siu-Ting K, Carrillo J. Two new harlequin frogs (Anura: *Atelopus*) from the Andes of northern Peru. Salamandra. 2008;44(3):163–76. PubMed PMID: BIOABS:BACD200800405425.

[23] Seimon TA, Seimon A, Daszak P, Halloy SRP, Schloegel LM, Aguilar CA, et al Upward range extension of Andean anurans and chytridiomycosis to extreme elevations in response to tropical deglaciation. Global Change Biology. 2007;13(1):288–99.

[24] Catenazzi A, Lehr E, Vredenburg VT. Thermal physiology, disease and amphibian declines in the eastern slopes of the Andes. Conservation Biology. 2014;28:509–17. doi: 10.1111/cobi.12194 24372791 10.1111/cobi.12194

[25] Kinney VC, Heemeyer JL, Pessier AP, Lannoo MJ. Seasonal pattern of *Batrachochytrium dendrobatidis* infection and mortality in *Lithobates areolatus*: Affirmation of Vredenburg's "10,000 zoospore rule". Plos One. 2011;6(3). doi: 10.1371/journal.pone.0016708 21423745 10.1371/journal.pone.0016708 PMC3053364

[26] Catenazzi A, Lehr E, von May R. The amphibians and reptiles of Manu National Park and its buffer zone, Amazon basin and eastern slopes of the Andes, Peru. Biota Neotropica. 2013;13(4):269–83.

[27] Burkart D, Flechas SV, Vredenburg VT, Catenazzi A. Cutaneous bacteria, but not peptides, are associated with chytridiomycosis resistance in Peruvian marsupial frogs. Animal Conservation. 2017; doi: 10.1111/acv.12352

[28] Garner TW, Garcia G, Carroll B, Fisher MC. Using itraconazole to clear *Batrachochytrium dendrobatidis* infection, and subsequent depigmentation of *Alytes muletensis* tadpoles. Diseases of Aquatic Organisms. 2009;83:257–60. doi: 10.3354/dao02008 19402457 10.3354/dao02008

[29] Catenazzi A, Vredenburg VT, Lehr E. *Batrachochytrium dendrobatidis* in the live frog trade of *Telmatobius* (Anura: Ceratophryidae) in the tropical Andes. Diseases of Aquatic Organisms. 2010;92(2–3):187–91. doi: 10.3354/dao02250 21268980 10.3354/dao02250

[30] Warne RW, LaBumbard B, LaGrange S, Vredenburg VT, Catenazzi A. Co-infection by chitrid fungus and Ranaviruses in wild and harvested frogs in the Tropical Andes. Plos One. 2016;11(1):e0145864 doi: 10.1371/journal.pone.0145864 26726999 10.1371/journal.pone.0145864 PMC4701007

[31] Boyle DG, Boyle DB, Olsen V, Morgan JAT, Hyatt AD. Rapid quantitative detection of chytridiomycosis (*Batrachochytrium dendrobatidis*) in amphibian samples using real-time Taqman PCR assay. Diseases of Aquatic Organisms. 2004;60(2):141–8. doi: 10.3354/dao060141 15460858 10.3354/dao060141

[32] Simmons JE. Herpetological collecting and collection management Ithaca, NY: Society for the Study of Amphibians and Reptiles; 2002. 153 p.

[33] Hyatt AD, Boyle DG, Olsen V, Boyle DB, Berger L, Obendorf D, et al Diagnostic assays and sampling protocols for the detection of *Batrachochytrium dendrobatidis*. Diseases of Aquatic Organisms. 2007;73(3):175–92. doi: 10.3354/dao073175 17330737 10.3354/dao073175

[34] Nicholson BJ. On the F-Distribution for calculating Bayes Credible Intervals for fraction nonconforming. IEEE Transactions on Reliability. 1985;R-34:227–8.

[35] Therneau TM. A Package for Survival Analysis in S. R package version 2.37–2. 2012. Available from: http://CRAN.R-project.org/package=survival.

[36] Therneau TM, Grambsch PM. Modeling Survival Data: Extending the Cox Model. New York: Springer; 2000.

[37] Wake DB, Vredenburg VT. Are we in the midst of the sixth mass extinction? A view from the world of amphibians. Proceedings of the National Academy of Sciences of the United States of America. 2008;105:11466–73. doi: 10.1073/pnas.0801921105 . PubMed PMID: WOS:000258561200003. 18695221 10.1073/pnas.0801921105 PMC2556420

[38] Fisher MC, Garner TWJ, Walker SF. Global emergence of *Batrachochytrium dendrobatidis* and amphibian chytridiomycosis in space, time, and host. Annual Review of Microbiology. 2009;63:291–310. doi: 10.1146/annurev.micro.091208.073435 19575560 10.1146/annurev.micro.091208.073435

[39] Grogan LF, Phillott AD, Scheele BC, Berger L, Cashins SD, Bell SC, et al Endemicity of chytridiomycosis features pathogen overdispersion. Journal of Animal Ecology. 2016;85(3):806–16. doi: 10.1111/1365-2656.12500 26847143 10.1111/1365-2656.12500

[40] Longo AV, Rodriguez D, Leite DdS, Toledo LF, Mendoza Almeralla C, Burrowes PA, et al ITS1 copy number varies among *Batrachochytrium dendrobatidis* strains: Implications for qPCR estimates of infection intensity from field-collected amphibian skin swabs. Plos One. 2013;8(3). doi: 10.1371/journal.pone.0059499 23555682 10.1371/journal.pone.0059499 PMC3605245

[41] Berger L, Speare R, Hines HB, Marantelli G, Hyatt AD, McDonald KR, et al Effect of season and temperature on mortality in amphibians due to chytridiomycosis. 2004;82(7):434–9. 15354853 10.1111/j.1751-0813.2004.tb11137.x

[42] Rowley JJL, Alford RA. Behaviour of Australian rainforest stream frogs may affect the transmission of chytridiomycosis. Diseases of Aquatic Organisms. 2007;77(1):1–9. doi: 10.3354/dao01830 17933392 10.3354/dao01830

[43] Raffel TR, Rohr JR, Kiesecker JM, Hudson PJ. Negative effects of changing temperature on amphibian immunity under field conditions. Functional Ecology. 2006;20(5):819–28. doi: 10.1111/j.1365-2435.2006.01159.x

[44] Raffel TR, Halstead NT, McMahon TA, Davis AK, Rohr JR. Temperature variability and moisture synergistically interact to exacerbate an epizootic disease. Proceedings Biological sciences / The Royal Society. 2015;282(1801). doi: 10.1098/rspb.2014.2039 PubMed PMID: MEDLINE:25567647. 25567647 10.1098/rspb.2014.2039 PMC4308995

[45] Carey C. Infectious disease and worldwide declines of amphibian populations, with comments on emerging diseases in coral reef organisms and in humans. Environmental Health Perspectives. 2000;108:143–50. doi: 10.2307/3454639 10698730 10.1289/ehp.00108s1143 PMC1637788

[46] Woodhams DC, Geiger CC, Reinert LK, Rollins-Smith LA, Lam B, Harris RN, et al Treatment of amphibians infected with chytrid fungus: learning from failed trials with itraconazole, antimicrobial peptides, bacteria, and heat therapy. Diseases of Aquatic Organisms. 2012;98(1):11–25. doi: 10.3354/dao02429 22422126 10.3354/dao02429

[47] Puschendorf R, Hoskin CJ, Cashins SD, McDonald K, Skerratt LF, Vanderwal J, et al Environmental refuge from disease-driven amphibian extinction. Conservation Biology. 2011;25(5):956–64. doi: 10.1111/j.1523-1739.2011.01728.x 21902719 10.1111/j.1523-1739.2011.01728.x

[48] Stice MJ, Briggs CJ. Immunization is ineffective at preventing infection and mortality due to the amphibian chytrid fungus *Batrachochytrium dendrobatidis*. Journal of Wildlife Diseases. 2010;46(1):70–7. doi: 10.7589/0090-3558-46.1.70 20090019 10.7589/0090-3558-46.1.70

[49] Cashins SD, Grogan LF, McFadden M, Hunter D, Harlow PS, Berger L, et al Prior infection does not improve survival against the amphibian disease chytridiomycosis. Plos One. 2013;8(2). doi: 10.1371/journal.pone.0056747 23451076 10.1371/journal.pone.0056747 PMC3579874

[50] Hudson MA, Young RP, Lopez J, Martin L, Fenton C, McCrea R, et al In-situ itraconazole treatment improves survival rate during an amphibian chytridiomycosis epidemic. Biological Conservation. 2016;195:37–45. doi: 10.1016/j.biocon.2015.12.041

[51] Geiger CC, Bregnard C, Maluenda E, Voordouw MJ, Schmidt BR. Antifungal treatment of wild amphibian populations caused a transient reduction in the prevalence of the fungal pathogen, *Batrachochytrium dendrobatidis*. Sci Rep. 2017;7 doi: 10.1038/s41598-017-05798-9 28729557 10.1038/s41598-017-05798-9 PMC5519715

[52] Farrer RA, Weinert LA, Bielby J, Garner TWJ, Balloux F, Clare F, et al Multiple emergences of genetically diverse amphibian-infecting chytrids include a globalized hypervirulent recombinant lineage. Proceedings of the National Academy of Sciences of the United States of America. 2011;108(46):18732–6. doi: 10.1073/pnas.1111915108 22065772 10.1073/pnas.1111915108 PMC3219125

[53] Voyles J, Cashins SD, Rosenblum EB, Puschendorf R. Preserving pathogens for wildlife conservation: a case for action on amphibian declines. Oryx. 2009;43(4):527–9. doi: 10.1017/s0030605309990469

[54] Scheele BC, Hunter DA, Banks SC, Pierson JC, Skerratt LF, Webb R, et al High adult mortality in disease-challenged frog populations increases vulnerability to drought. Journal of Animal Ecology. 2016;85(6):1453–60. doi: 10.1111/1365-2656.12569 27380945 10.1111/1365-2656.12569

