# Supplementary material for: Epizootic to enzootic transition of a fungal disease in tropical Andean frogs: Are surviving species still susceptible?
Source: PLoS One. 2017 Oct 17;12(10):e0186478. doi: 10.1371/journal.pone.0186478 (PMC5645123; doi:10.1371/journal.pone.0186478)
Supplement: S1 Table — This is the S1 Fig legend. (DOCX) [file pone.0186478.s001.docx]

**Supplemental materials: Epizootic to enzootic transition of a fungal disease in tropical Andean frogs: are surviving species still susceptible?**

Alessandro Catenazzi, Andrea Swei, Jacob Finkle, Emily Foreyt, Lauren Wyman, Vance T. Vredenburg

**S1 Table.** Raw data for susceptibility trials. Abbreviations: ind = unique identifier for each individual; death = number of days alive; status at end of experiment: 0 = alive, 1 = dead; weight = initial body mass (g); telmasource = individual(s) Telmatobius used as source of infection; telmaze = infection intensity of Telmatobius source at time of purchase (ZE); postitraze = infection intensity on day following the end of itraconazol treatment.

*Gastrotheca excubitor* (duration: 26 days)

| ind | death | status | weight | group | telmasource | telmaze | postitraze |
| --- | --- | --- | --- | --- | --- | --- | --- |
| 266 | 26 | 0 | 7.6 | control | NA | 0 | 9.17E-03 |
| 267 | 26 | 0 | 8.1 | control | NA | 0 | 400 |
| 329 | 26 | 0 | 3.6 | control | NA | 0 | 46.48 |
| 335 | 26 | 0 | 1.2 | control | NA | 0 | 0 |
| 264 | 26 | 0 | 5.1 | control | NA | 0 | 4.52 |
| 265 | 26 | 0 | 0.8 | control | NA | 0 | 2.384 |
| 386 | 26 | 0 | 4 | control | NA | 0 | 4.896 |
| 330 | 26 | 0 | 2.1 | infected | t415 | 3824.8 | 268 |
| 383 | 26 | 0 | 3.9 | infected | t392x3.t414x2 | 5730.24 | 0 |
| 384 | 26 | 0 | 5.8 | infected | t389 | 236 | 3.568 |
| 385 | 26 | 0 | 4 | infected | t393 | 732 | 0 |
| 387 | 26 | 0 | 5.4 | infected | t395x4.t417 | 742.896 | 0 |

*Gastrotheca nebulanastes* (duration: 51 days)

| ind | death | status | weight | group | telmasource | telmaze | postitraze |
| --- | --- | --- | --- | --- | --- | --- | --- |
| 11 | 5 | 1 | 3 | infected | t74 | 693.6 | 0 |
| 12 | 34 | 1 | 3.2 | infected | t79 | 0 | 0 |
| 13 | 51 | 0 | 0.4 | infected | t73 | 8.96 | 1.536 |
| 17 | 47 | 1 | 2.8 | infected | t69 | 21.52 | 0 |
| 18 | 48 | 1 | 7.2 | infected | t76x3.t73x2 | 2596.064 | 0 |
| 19 | 47 | 1 | 2.9 | infected | t68 | 28.64 | 0 |
| 8 | 51 | 0 | 6.8 | control | NA | 0 | 0 |
| 6 | 51 | 0 | 3.3 | control | NA | 0 | 0 |
| 14 | 51 | 0 | 4.5 | control | NA | 0 | 0 |
| 15 | 51 | 0 | 2.3 | control | NA | 0 | 0 |
| 39 | 51 | 0 | 3.3 | control | NA | 0 | 0 |

*Hypsiboas gladiator* (duration: 33 days)

| ind | death | status | weight | group | telmasource | telmaze | postitraze |
| --- | --- | --- | --- | --- | --- | --- | --- |
| 198 | 13 | 1 | 6.6 | control | NA | 0 | 1.14E+00 |
| 199 | 26 | 1 | 4.7 | control | NA | 0 | 2.688 |
| 200 | 33 | 0 | 2 | control | NA | 0 | 0 |
| 216 | 33 | 0 | 3.9 | control | NA | 0 | 1.432 |
| 289 | 33 | 0 | 3.6 | control | NA | 0 | 0 |
| 195 | 33 | 0 | 3.5 | infected | t372 | 51.2 | 0 |
| 196 | 13 | 1 | 6.3 | infected | t380 | 171.2 | 0 |
| 197 | 13 | 1 | 3.4 | infected | t376x4.t371 | 34.048 | 0 |
| 217 | 27 | 1 | 5.7 | infected | t171 | 375.2 | 0 |
| 288 | 19 | 1 | 4.6 | infected | t367 | 0 | 0 |

*Psychrophrynella usurpator* (duration: 52 days)

| ind | death | status | weight | group | telmasource | telmaze | postitraze |
| --- | --- | --- | --- | --- | --- | --- | --- |
| 21 | 52 | 0 | 0.8 | control | NA | 0 | 0 |
| 33 | 52 | 0 | 0.3 | control | NA | 0 | 0 |
| 37 | 52 | 0 | 0.6 | control | NA | 0 | 0 |
| 38 | 52 | 0 | 0.6 | control | NA | 0 | 0 |
| 44 | 52 | 0 | 0.5 | control | NA | 0 | 0 |
| 1 | 52 | 0 | 0.6 | infected | t74 | 693.6 | 0 |
| 3 | 52 | 0 | 0.4 | infected | t66 | 0 | 0 |
| 23 | 52 | 0 | 0.8 | infected | t81 | 0.896 | 0 |
| 24 | 52 | 0 | 0.6 | infected | t64 | 0 | 0 |
| 26 | 52 | 0 | 0.2 | infected | t75 | 19.92 | 0 |
| 27 | 52 | 0 | 0.9 | infected | t77 | 1353.6 | 0 |
| 28 | 52 | 0 | 0.3 | infected | t78 | 603.2 | 0 |
| 29 | 52 | 0 | 0.9 | infected | t73 | 8.96 | 0 |
| 30 | 52 | 0 | 0.9 | infected | t79 | 0 | 0 |
| 32 | 52 | 0 | 0.6 | infected | t63 | 181.6 | 0 |
| 35 | 52 | 0 | 1.1 | infected | t70x4+t75 | 1964.944 | 0 |
| 36 | 52 | 0 | 0.6 | infected | t68 | 28.64 | 0 |
| 45 | 52 | 0 | 0.8 | infected | t76 | 4320.8 | 0 |
| 46 | 52 | 0 | 0.8 | infected | t69 | 21.52 | 0 |

*Pristimantis danae* (duration: 33 days)

| ind | death | status | weight | group | telmasource | telmaze | postitraze |
| --- | --- | --- | --- | --- | --- | --- | --- |
| 97 | 33 | 0 | 0.8 | control | NA | 0 | 0 |
| 206 | 33 | 0 | 2.1 | control | NA | 0 | 0 |
| 259 | 33 | 0 | 0.6 | control | NA | 0 | 0 |
| 262 | 33 | 0 | 1.3 | control | NA | 0 | 0 |
| 284 | 33 | 0 | 4.9 | control | NA | 0 | 0 |
| 92 | 24 | 1 | 0.4 | infected | t368 | 0 | 9.92 |
| 96 | 33 | 0 | 2.6 | infected | t372 | 51.2 | 4.616 |
| 203 | 33 | 0 | 2.3 | infected | t171 | 375.2 | 0 |
| 204 | 33 | 0 | 9.1 | infected | t175 | 1905.6 | 0 |
| 205 | 30 | 1 | 5.2 | infected | t381 | 142.4 | 0 |
| 207 | 33 | 0 | 2.1 | infected | t367 | 0 | 0 |
| 258 | 33 | 0 | 1.2 | infected | t380 | 171.2 | 0 |
| 260 | 33 | 0 | 1.6 | infected | t376 | 42.56 | 0 |
| 261 | 26 | 1 | 1.4 | infected | t374 | 155.2 | 0.6368 |
| 281 | 33 | 0 | 1.1 | infected | t373 | 299.2 | 0 |
| 283 | 33 | 0 | 2.8 | infected | t370 | 4.056 | 0 |
| 285 | 33 | 0 | 1.1 | infected | t371 | 0 | 0 |
| 286 | 29 | 1 | 1 | infected | t366 | 0.1088 | 8.64 |
| 287 | 33 | 0 | 0.9 | infected | t379x3.t378x2 | 30.08 | 0 |

*Pristimantis pharangobates* (duration: 27 days)

| ind | death | status | weight | group | telmasource | telmaze | postitraze |
| --- | --- | --- | --- | --- | --- | --- | --- |
| 41 | 24 | 1 | 0.7 | infected | t380x3.t419x2 | 245.12 | 0 |
| 162 | 18 | 1 | 0.3 | infected | t169 | 1428.8 | 0 |
| 164 | 20 | 1 | 0.4 | infected | t381 | 142.4 | 0 |
| 291 | 27 | 0 | 2.6 | control | NA | 0 | 6.536 |
| 352 | 27 | 0 | 0.9 | control | NA | 0 | 0 |
| 354 | 27 | 0 | 0.7 | control | NA | 0 | 0 |
| 362 | 27 | 0 | 1.2 | control | NA | 0 | 0 |
| 365 | 27 | 0 | 1.7 | control | NA | 0 | 0.1808 |
| 42 | 27 | 0 | 4.4 | infected | t367x4.t418.1 | 284.8 | 0 |
| 43 | 27 | 0 | 0.7 | infected | t388 | 514.4 | 0 |
| 165 | 27 | 0 | 0.3 | infected | t390x3.t421 | 11830.72 | 5.496 |
| 290 | 27 | 0 | 0.8 | infected | t175x3.t414x2 | 1428.48 | 0 |
| 292 | 27 | 0 | 1.1 | infected | t171x2.t395x3 | 703.52 | 0 |
| 347 | 27 | 0 | 0.5 | infected | t372x2.t414x3 | 448.16 | 0 |
| 349 | 27 | 0 | 0.7 | infected | t378x3.t399x2 | 1475.84 | 10.08 |
| 355 | 27 | 0 | 1 | infected | t370x2.t418x3 | 215.224 | 0 |
| 356 | 27 | 0 | 0.9 | infected | t366x2.t398.t417x2 | 626.7955 | 3.248 |
| 357 | 27 | 0 | 1.2 | infected | t413x3.t418x2 | 8016.8 | 22.24 |
| 358 | 27 | 0 | 0.7 | infected | t368x2.t392x3 | 5445.12 | 15.12 |
| 359 | 27 | 0 | 1 | infected | t419 | 25541.6 | 8.4 |
| 360 | 27 | 0 | 0.8 | infected | t393 | 732 | 4.544 |
| 361 | 27 | 0 | 1.1 | infected | t415 | 3824.8 | 0 |
| 363 | 27 | 0 | 1.7 | infected | t397x3.t389.t392 | 2004.8 | 0 |
| 364 | 27 | 0 | 3.4 | infected | t417 | 24.88 | 0 |

*Pristimantis platydactytlus* (duration: 29 days)

| ind | death | status | weight | group | telmasource | telmaze | postitraze |
| --- | --- | --- | --- | --- | --- | --- | --- |
| 147 | 29 | 0 | 1 | control | NA | 0 | 2.07E-02 |
| 211 | 29 | 0 | 0.8 | control | NA | 0 | 1.064 |
| 229 | 29 | 0 | 0.4 | control | NA | 0 | 0.46894 |
| 350 | 29 | 0 | 0.5 | control | NA | 0 | 1.41693 |
| 86 | 27 | 1 | 1 | infected | t367 | 0 | 0 |
| 166 | 29 | 0 | 0.7 | infected | t366x2.t395x3 | 553.4835 | 0 |
| 210 | 29 | 0 | 0.7 | infected | t372x3.t420x2 | 2367.36 | 0 |
| 231 | 29 | 0 | 0.5 | infected | t171x4.t415 | 1065.12 | 0 |
| 245 | 17 | 1 | 1 | infected | t169 | 1482.8 | 1.02857 |
| 246 | 8 | 1 | 1.2 | infected | t371 | 0 | 0.01037 |
| 247 | 29 | 0 | 0.3 | infected | t175x4.t389 | 1571.68 | 0.00306 |
| 251 | 15 | 1 | 1.4 | infected | t378x3.t390x2 | 1780.8 | 0 |
| 277 | 29 | 0 | 0.3 | infected | t399x4.t395 | 3045.92 | 0 |
| 278 | 18 | 1 | 0.3 | infected | t414x4.t370 | 571.0512 | 0.27231 |
| 280 | 13 | 1 | 0.2 | infected | t381x4.t393 | 260.32 | 1.85709 |
| 351 | 29 | 0 | 0.6 | infected | t368 | 0 | 0 |
| 353 | 29 | 0 | 1.1 | infected | t380x4.t417 | 141.936 | 1.55291 |

*Pristimantis toftae* (duration: 31 days)

| ind | death | status | weight | group | telmasource | telmaze | postitraze |
| --- | --- | --- | --- | --- | --- | --- | --- |
| 100 | 22 | 1 | 1.2 | infected | t381 | 142.4 | 0 |
| 108 | 22 | 1 | 1.8 | infected | t373 | 299.2 | 0 |
| 253 | 21 | 1 | 2.3 | infected | t366 | 0.1088 | 0 |
| 256 | 25 | 1 | 1.4 | infected | t379 | 171.2 | 0 |
| 104 | 31 | 0 | 1.8 | infected | NA | 0 | 0 |
| 87 | 31 | 0 | 0.6 | control | NA | 0 | 0 |
| 106 | 31 | 0 | 0.7 | control | NA | 0 | 0 |
| 201 | 31 | 0 | 2 | control | NA | 0 | 22.4 |
| 252 | 31 | 0 | 0.9 | control | NA | 0 | 0.348 |
| 254 | 31 | 0 | 1.8 | control | NA | 0 | 0 |
