# Supplementary material for: Epizootic to enzootic transition of a fungal disease in tropical Andean frogs: Are surviving species still susceptible?
Source: PLoS One. 2017 Oct 17;12(10):e0186478. doi: 10.1371/journal.pone.0186478 (PMC5645123; doi:10.1371/journal.pone.0186478)
Supplement: S1 File — Videos of diseased individuals used during the experiments. (DOCX) [file pone.0186478.s002.docx]

**Supplemental materials: Epizootic to enzootic transition of a fungal disease in tropical Andean frogs: are surviving species still susceptible?**

Alessandro Catenazzi, Andrea Swei, Jacob Finkle, Emily Foreyt, Lauren Wyman, Vance T. Vredenburg

**S1 File. Videos showing symptoms of chytridiomycosis in highly infected frogs used in the susceptibility trials.** Videos of diseased individuals used during the experiments.

*Pristimantis toftae* (individual 108.12): <http://bit.ly/2h7Y22W>

*Telmatobius marmoratus*: <http://bit.ly/2fe725P>
