# Supplementary material for: Epizootic to enzootic transition of a fungal disease in tropical Andean frogs: Are surviving species still susceptible?
Source: PLoS One. 2017 Oct 17;12(10):e0186478. doi: 10.1371/journal.pone.0186478 (PMC5645123; doi:10.1371/journal.pone.0186478)
Supplement: S2 Table — “–”indicates that no data are available for a given survey and species; Table 2 column refers to data (population ratios and results of GLM analyses) reported in Table 2. When data from both sampling techniques are available, results from leaf litter plots are reported in Table 2 of the manuscript. (DOCX) [file pone.0186478.s003.docx]

**Supplemental materials: Epizootic to enzootic transition of a fungal disease in tropical Andean frogs: are surviving species still susceptible?**

Alessandro Catenazzi, Andrea Swei, Jacob Finkle, Emily Foreyt, Lauren Wyman, Vance T. Vredenburg

**S2 Table. P-values from generalized linear models for change in relative abundances before (1998–1999) and after the epizootic (2008–2009) for leaf litter plots and visual surveys.** “–“ indicates that no data are available for a given survey and species; Table 2 column refers to data (population ratios and results of GLM analyses) reported in Table 2. When data from both sampling techniques are available, results from leaf litter plots are reported in Table 2 of the manuscript.

**Species Leaf litter Surveys Reported Table 2**

**Pop. ratio *P* Pop. ratio *P***

*Gastrotheca excubitor*  0.54 0.113 – – Leaf litter

*Gastrotheca nebulanastes*  – – **0.07 <0.001**  Visual surveys

*Hypsiboas gladiator*  – – 0.51 0.876 Visual surveys

*Pristimantis danae* 0.66 0.206 0.63 0.143 Leaf litter

*P. pharangobates*  0.59 0.088 0.45 0.168 Leaf litter

*P. platydactylus*  – – 0.45 0.433 Visual surveys

*P. toftae*  0.51 0.083 0.48 0.154 Leaf litter

*Psychrophrynella usurpator* 2.24 0.147 – – Leaf litter
